# Current Challenges and Achievements in Maternal Immunization Research

**DOI:** 10.3389/fimmu.2018.00436

**Published:** 2018-03-06

**Authors:** Flor M. Munoz

**Affiliations:** ^1^Department of Pediatrics, Baylor College of Medicine, Houston, TX, United States; ^2^Department of Molecular Virology and Microbiology, Baylor College of Medicine, Houston, TX, United States

**Keywords:** maternal immunization, ethics, research, pregnancy, inclusion, regulatory, vaccination

## Abstract

Maternal immunization has the potential to significantly improve maternal and child health worldwide by reducing maternal and infant morbidity and mortality associated with disease caused by pathogens that are particularly relevant in the perinatal period and in early life, and for which no alternative effective preventive strategies exist. Research on all aspects related to vaccines for administration during pregnancy is ongoing with support of multiple stakeholders and global participation. Substantial progress has been made, and the availability of new vaccines licensed exclusively for use in pregnant women to protect their infants has become an achievable goal. This review provides an update of the current challenges and achievements in maternal immunization research, focusing on recent milestones that advance the field and the prospects to make maternal immunization a feasible and accessible strategy to improve global health.

## Introduction

The goal of maternal immunization is to boost maternal levels of specific antibodies to provide the newborn and young infant with sufficient IgG antibody concentrations at birth to protect them against infections occurring during a period of increased vulnerability, until they are able to adequately respond to their own active immunizations or infectious challenges. Newborns and young infants are at greatest risk of morbidity and mortality from infectious diseases, and they depend of maternal antibodies to resist these infections in early life. Maternal antibodies can be optimized during pregnancy given that pregnant women have intact humoral immune responses to vaccines and adequately produce antibodies, which can be efficiently transferred to the fetus through an active receptor-mediated transport system in the placenta. Higher concentrations of antibody at birth result in protection from infection and disease, or in delayed onset and decreased severity of various infectious diseases in the newborn. Examples of this concept include passive maternal antibody protection against tetanus, pertussis, respiratory syncytial virus (RSV), influenza virus, and group B streptococcus (GBS) infections, among others.

Research on maternal immunization is not new; as vaccines were developed, their administration to pregnant mothers to protect them and/or their infants was considered and evaluated, including protection against small pox with vaccinia vaccine in the late 1800s, whole cell pertussis vaccine (DTP) in the 1940s, influenza vaccine after the 1950s pandemics, and tetanus toxoid vaccine to prevent maternal and neonatal tetanus worldwide since the 1960s. Despite the success of the Maternal–Neonatal Tetanus Elimination program of the World Health Organization (WHO) (http://www.who.int/immunization/diseases/MNTE_initiative/en/), there was a paucity of active research on maternal immunization for several years in the twentieth century, in part due to concerns with the safety of administering any drug or biologic to women during pregnancy, particularly after the experience of the drug thalidomide in the 1960s, which was associated with severe limb and other deformities in infants born to women who took this unlicensed medication in the US to treat hyperemesis gravidarum.

## Key Issues on Maternal Immunization Research

The potential impact of maternal immunization as a public strategy to prevent disease in mothers and infants is well recognized. Yet, there are no vaccines currently approved or licensed specifically for use in pregnant women. Licensed vaccines that are recommended for non-pregnant adults may be administered to pregnant women based on need and a risk:benefit assessment. When the risk of exposure and disease from a vaccine preventable infection is high for a mother and/or her fetus, and an effective vaccine is available, the benefit of the vaccine protection is greater than any potential theoretical risk from the vaccine, which is in turn considered to be lower than the risk of acquiring the infection and disease the vaccine can prevent. Licensed vaccines that have not been formally evaluated in or approved for pregnant women are therefore recommended for administration during pregnancy by the WHO and the US Centers for Disease Control and Prevention (CDC), as well as local organizations in many countries (1, 2) (Table 1). These recommendations have evolved over time, and they differ in that the current WHO recommendations do not specifically recommend pertussis vaccination during pregnancy, except when there is a known high burden of disease, as implemented in several countries such as Canada and Australia; while CDC and other Public Health programs such as in the UK, recommend routine vaccination of all pregnant women with the tetanus, diphtheria, and reduced acellular pertussis antigen content (Tdap) vaccine for all women, at every pregnancy. The specific timing of administration of this vaccine is also variable in different countries. Similarly, while tetanus vaccination is recommended for all pregnancies by WHO, most industrialized countries in Europe and North America, where pediatric vaccination coverage is high and the risk of tetanus infection at birth is negligible, do not routinely recommend tetanus vaccine administration during pregnancy. It is only given now because of the use of Tdap. Finally, influenza vaccination during pregnancy is considered an essential element of prenatal care in the US, and pregnant women have one of the highest influenza vaccination coverage rates in this country. However, while pregnant women are not excluded from influenza vaccination, routine administration is not the standard in most countries.

**Table 1:** Recommended vaccines for maternal immunization [World Health Organization (WHO)].

Generally recommended	Recommended for disease prevention in specific situations	Contraindicated
Tetanus (TT, Td)Influenza inactivated^a^Acellular pertussis vaccine (Tdap) only in areas of burden^b^	CholeraYellow feverMeningitis A (meningococcal)Hepatitis A, B, and E, Japanese encephalitisPolio (OPV and IPV)Rabies	BCGMeaslesMumpsRubellaVaricellaLive typhoid T21aLive influenza

*^a^Influenza vaccine is recommended by WHO for administration in pregnant women in regions where influenza vaccine programs are already in place. Influenza vaccination is recommended as part of routine antenatal care in the US and several countries in Latin America*.

*^b^Tdap is routinely recommended for pregnant women in the US, the UK, some provinces of high burden in Europe, Canada, and Australia, as well as several countries in Latin America*.

Given that currently licensed vaccines are not specifically indicated for pregnant women, there might be reluctance by some providers and government agencies worldwide to recommend routine vaccination in this population. However, the US Federal Drug Administration (FDA) addresses this concern by approving labeling clearly stating in that licensed vaccines that are recommended for pregnant women (such as influenza and Tdap) are NOT contraindicated for use in pregnant women, and specific considerations regarding safety of use during pregnancy are addressed in the pregnancy subsection of the FDA approved labeling (3). Furthermore, the safety of these vaccines continues to be monitored through post-licensure surveillance mechanisms, such as pregnancy registries and large passive and active adverse event reporting and surveillance systems (4).

Ensuring and evaluating the safety of vaccines administered to pregnant women is a key component of any maternal immunization program or recommendation. This is particularly true now that new vaccines that can benefit pregnant women and their infants are being developed, such as vaccines to protect against GBS and RSV. An important issue is the need for harmonization of standard definitions of key safety outcomes after maternal vaccination and of a systematic approach to the assessment of safety throughout the life cycle of a vaccine, but particularly after implementation as large number of pregnant women are vaccinated. It is critical to consider the inherent risks associated with pregnancy itself, and to clearly understand the background rate of these risks in specific populations. Furthermore, to evaluate the impact of maternal immunization as a public health strategy to impact the burden of morbidity and mortality associated with the infection it prevents, it is necessary to establish baseline rates of these outcomes to demonstrate the efficacy and benefit of the vaccines in both mothers and infants. Finally, the ethical and regulatory aspects surrounding the inclusion of pregnant women as research subjects also influence the progress of the development of vaccines for maternal immunization.

## Recent Milestones in Maternal Immunization Research

Substantial progress has occurred in maternal immunization research (Table 2). Maternal immunization research has been supported by National Institutes of Health in the US for decades, spanning basic science, clinical, epidemiological, and translational research (5). Studies of relevant pathogens, including GBS, *Haemophilus influenzae* type b, *Streptococcus pneumoniae*, and tetanus were conducted during the 1980s and 1990s; studies of pertussis and RSV were prioritized from the 1990s to the first decade of the twenty-first century, while studies of seasonal and pandemic influenza vaccine studies have been conducted continuously for 40 years. Experimental and licensed vaccines for these pathogens were evaluated in phase I/II clinical trials in pregnant women under contract with various public and academic institutions in the US. Furthermore, these programs promoted research related to maternal immunization from vaccine antigen identification to the development of pertinent laboratory assays and reference materials, as well as animal models and developmental toxicity studies, and epidemiology and safety studies. In 2013, guidance documents on research, protocol design, and assessment of safety of vaccines during pregnancy were developed (6, 7). Other guidance documents have since been published, providing a framework for the study of vaccines and other biologics in pregnant women.

**Table 2:** Milestones in the development of vaccines for maternal immunization.

Time period	Milestones
1940s	Studies of whole cell pertussis vaccine (DTPw) in pregnant women to protect infants in the US (8)
1960s	Influenza vaccines recommended for pregnant women, considered a high risk group for influenza complications after the 1957 pandemic (8) Maternal immunization with tetanus toxoid demonstrated to prevent neonatal tetanus in clinical study in Papua New Guinea (9)
1970s	Tetanus toxoid added to World Health Organization (WHO) Expanded Program on Immunization (10)
1980s	Maternal–Neonatal Tetanus Elimination program goal set by the WHO (10) Phase I/II studies of vaccines in pregnant women and various studies related to maternal immunization supported by NIH are initiated in the US (5)
1990s	Phase I/II studies of vaccines in pregnant women and various studies related to maternal immunization supported by NIH are ongoing in the US (5) Influenza vaccine is routinely recommended for pregnant women in the US, regardless of underlying medical conditions (11)
2000s	NIH clinical studies of vaccines in pregnancy continue (5) Brighton Collaboration is formed (12) WHO supports influenza vaccine recommendations in pregnancy (13) Study in Bangladesh demonstrates efficacy of influenza vaccine given to pregnant women in protecting mothers and infants against laboratory confirmed influenza illness (14) The Bill and Melinda Gates Foundation supports 3 large studies of influenza maternal immunization in Nepal, Mali, and South Africa (15–17) MenAfrivac program in the African meningitis belt does not exclude pregnant women from receiving the meningococcal A vaccine (18) The 2009 influenza pandemic results in prioritization of maternal immunization research in the US and worldwide (19)
2010 to date	Publications of NIH guidance on Maternal Immunization Research and Toxicity Tables for pregnant women (7) GAIA is formed in response to call from WHO to work toward harmonization of the assessment of safety of vaccines in pregnancy (20) The WHO’s Strategic Advisory Group of Experts recommends influenza vaccination of pregnant women in countries were influenza vaccines are routinely administered (21) Given the reemergence of pertussis and infant mortality, maternal immunization with Tdap is recommended in the US and the UK in 2012 and subsequently other countries (22, 23) Safety and effectiveness data from the UK and the US continue to support the administration of Tdap for pregnant women (24–26) Research and health regulations support the inclusion of pregnant women in research (27–33) Multiple studies of vaccines for pregnant women are being conducted globally with the support of various stakeholders, including vaccines for the prevention of respiratory syncytial virus and group B streptococcus (19, 34–38)

In 2008, a pivotal study conducted in Bangladesh was published (14). This study demonstrated for the first time that maternal vaccination with influenza vaccine can protect mothers and their infants from laboratory confirmed influenza illness, with an efficacy in preventing infant influenza of 63%, similar to that achieved with active immunization. This study led to the support to three large studies of influenza vaccination of pregnant women by the Bill and Melinda Gates Foundation, conducted in Nepal, Mali, and South Africa. These seminal studies have now been completed, contributing significantly to the knowledge of the benefits and safety of influenza vaccination of mothers and infants, including HIV infected women, and providing critical information to guide decisions and policies surrounding maternal immunization (15–17). One important contribution of these trials was the determination of the relatively limited duration of protection of infants provided by maternally derived antibody, which decreased substantially after the second month of life (39). The 2009–2010 influenza pandemic was another critical event that resulted in the subsequent prioritization of maternal immunization research in the US and worldwide. The number of clinical trials and publications on the topic of maternal immunization has increased substantially since the pandemic. Importantly, the knowledge gained in aspects related to safety, immunogenicity, and implementation of influenza vaccines for pregnant women has resulted in more advances in this field than ever. An example of this was the acquisition of data on the safety and effectiveness of adjuvanted influenza vaccines in pregnant women (40). In general, there is a need for more immunogenic vaccines for use in all populations, including pregnant women, to improve effectiveness and further reduce the impact of influenza.

In 2012, prompted by evidence of reemergence of pertussis disease and associated infant mortality, maternal immunization with Tdap was recommended in the US and the UK as the most immediate and direct intervention to decrease pertussis in the first few months of life (22). Several other countries with high burden of pertussis disease in the Americas, Europe, and Australia also adopted this recommendation. Importantly, research on maternal immunization with Tdap flourished, filling critical gaps of information, such as understanding the optimal timing for maternal vaccination in the second trimester of gestation to achieve higher antibody concentrations in infants at birth, and better and longer duration of protection in the first few months of life until active immunization with pertussis containing vaccines is achieved (41). Another relevant concept associated with the utilization of Tdap vaccine in pregnancy is the potential blunting of infant immune responses to active immunization when high concentrations of maternal antibodies are present. This has been observed and documented for various antigens in the pertussis vaccines, including pertussis toxin, filamentous hemagglutinin, and pertactin, but relatively lower concentrations of vaccine-specific antibodies in infants after primary vaccination have not been associated with increased incidence or severity of pertussis disease in infants of vaccinated mothers, and preservation of priming and memory immune responses has been documented (42–45). Furthermore, the safety and effectiveness of the Tdap maternal immunization program have been demonstrated in the US and the UK, supporting continuation of this intervention in these countries (23–26). Similar programs are in place now in Latin America and other countries and regions with high burden of pertussis disease.

Currently, several studies are ongoing assessing various aspects of the use of licensed vaccines such as influenza and pertussis in pregnant women, as well focusing on the development of new vaccines specifically designed for administration during pregnancy, for the protection of infants against RSV and GBS in early life. Numerous RSV and GBS vaccines are in various phases of development, from preclinical to clinical trials, supported by multiple stakeholders from industry to private and public organizations (34–36). One RSV vaccine is currently in phase III of clinical development, promising, if successful, to be the first vaccine developed and licensed for specific use in pregnancy. Achieving this milestone has the potential to positively impact and change the landscape and practical applicability of infant disease prevention through maternal immunization. In addition to research focused on basic placental biology and immunology, understanding the role of passive and breast milk antibodies in infant protection and responses to natural infection and active immunization, and determining how to optimize maternal intervention to improve its safety and efficacy, other aspects that require further study include those related to acceptance, feasibility, and logistics of implementation of maternal immunization in different settings and populations. Furthermore, aspects related to education of mothers and providers, utilization, communications, and long-term surveillance and assessment of vaccine safety are paramount for the success of maternal immunization as a public health strategy to improve maternal and child globally. The field of maternal immunization research is therefore open, active, and rich.

## Progress in the Regulatory Aspects Related to Maternal Immunization and Research

The perception of risk of any intervention during pregnancy has evolved over time. Before the demonstration that the use of thalidomide during pregnancy was associated with birth defects, there were relatively little restrictions to what pregnant women were exposed to (46). This tragic association resulted in a shift toward strict restrictions of what pregnant women could be exposed to, including medications and vaccines, and the exclusion of pregnant women from research. However, there has been a culture change in recent years, driven by the need to develop effective immunization strategies and understanding that pregnant women and their infants can actually benefit from participating in clinical research. Their participation in clinical trials of vaccines and therapeutics ultimately will reduce any potential harm of these products, by generating useful information that is specifically relevant to pregnancy, and avoiding exclusion of women from receipt of potentially beneficial interventions available to the rest of the population. Having access to the benefits of participating in research and the results of this research will promote and improve maternal, fetal, and infant health. Clinical studies in pregnant women are carefully designed to minimize the risks of the intervention, particularly the risk to the fetus, and to balance the risk of participating in research with the risk of not having a potentially beneficial intervention available for mothers and infants.

Several recent milestones have been reached in the regulatory aspects of the assessment of vaccines for use in pregnancy (27). It is clear that for both novel vaccines, as well as for currently licensed vaccines not previously evaluated in pregnant women, regulatory agencies approval for use during pregnancy would result in inclusion of specific information in the product label that would facilitate the acceptance and use of the vaccine by health-care providers and the public in general. One important step toward facilitating the utilization of vaccines in pregnancy is the recent update to the US FRA pregnancy and lactation labeling rule, whereby product label pregnancy risk categories designated with letters as A, B, C, D, and X that were difficult to put into practice have been replaced with a narrative descriptions of the risks of using the vaccine during pregnancy, as informed by any source of information, including both observational and prospective studies (28). In 2015, vaccine manufacturers sought guidance from the Vaccines and Related Biological Products Advisory Committee of the FDA to work toward the development of vaccines for maternal immunization. In their fall meeting, the determination was made that the regulatory approval process of vaccines indicated for maternal immunization to prevent infant disease would be guided by regulations outlined in Title 21 of the Code of Federal Regulations and standards set forth in applicable documents such as the ICH guidelines and FDA guidance documents (29). The groups agreed that the path to development and licensure of a vaccine for pregnant women would be product specific and designed to support the indication being sought. Key aspects to consider would include the use of serologic endpoints as markers of passive protection in the infants, the evaluation of duration of immunity and immune interference with childhood vaccines, and the duration and type of safety follow-up. Importantly, the committee considered that observational studies could be used as an approach to confirm the effectiveness of already licensed vaccines that are recommended for use in pregnancy in the US.

Progress has also been made in regulations that further expand the options for pregnant women to be included in research. The updated “Common Rule,” which is the set of federal regulations for the ethical conduct of human subject research in the US, clearly delineates that pregnant women or fetuses may be involved in research if several conditions are met, including the prior conduct, when scientifically appropriate, of preclinical studies, including studies on pregnant animals (such as reproductive toxicology studies), and clinical studies, including studies on non-pregnant women (30). The document also delineates the risk categories for research based on the prospect of benefit for the women or the fetus, indicating that the risk of the research needs to be balanced with the prospect for benefit for the women OR the fetus, or if there is no such prospect of benefit, the risk to the fetus should be not greater than minimal when the purpose of the research is the development of important biomedical knowledge which cannot be obtained by any other means. The pregnant mother is given the right to provide consent for herself and for her baby, unless the prospect of direct benefit is solely to the fetus, then the consent of the pregnant mother and the father should be obtained, with exceptions allowed based on specific situations that would prevent the father from signing. These provisions help guide the Institutional Review Boards in their decision making regarding the participation of pregnant women in research.

Other advances relate to the change in classification of pregnant women from being considered a “vulnerable” population for research, to no longer being considered “vulnerable.” This challenge for maternal immunization was addressed by the National Vaccine Advisory Committee to the Department of Health and Human Services, who also recommended the prioritization of maternal immunization as a public health strategy, and the investment in the development of vaccines for pregnant women (31). Globally, the 2017 updated International Guidelines for Health-Related Research Involving Humans of the Council for International Organizations of Medical Sciences, in collaboration with the WHO, also conclude that women must be included in health-related research, unless a good scientific reason justifies their exclusion, and that women should provide informed consent for themselves (32). Finally, the 21st Century Cures Act, as law enacted by the US Congress in December 2016 and designed to help accelerate medical product development and faster access to patients to innovations, established a task force on research specific to pregnant women and lactating women, to provide advice and guidance to the Secretary of HHS, to address gaps in knowledge and research regarding safe and effective therapies for pregnant and lactating women, and authorized substantial funds for this task (33). A key provision of this law was the inclusion of vaccines administered during pregnancy in the Vaccine Injury Compensation Program, thereby providing coverage for claims of potential vaccination adverse effects on the fetus and the mother, for providers who administer vaccines to pregnant women. Specifically, the law states that “…both a woman who received a covered vaccine while pregnant and any child who was *in utero* at the time such woman received the vaccine shall be considered persons to whom the covered vaccine was administered and persons who received the covered vaccine.” This provision is a tremendous step toward the improvement of acceptance, confidence, and coverage of maternal immunization in the US.

## Efforts in Harmonization in the Assessment of Safety of Maternal Vaccines

In addition to the work of the NIH and investigators involved in maternal immunization research, one of the organizations that provided early contributions toward the goal of developing a consensus and harmonized assessment of the safety of vaccines during pregnancy is the Brighton Collaboration. This independent and non-profit partnership was formed in the year 2000 as a voluntary international group seeking to facilitate the development, evaluation, and dissemination of high quality information about the safety of human vaccines. The group stated by developing a common language and standardized research methods to improve the accuracy and consistency of vaccine risk assessment. In 2014, stemming from a call by WHO, and with support from the Bill and Melinda Gates Foundation, the GAIA (Global Alignment on Immunization Safety Assessment in pregnancy) consortium was formed, with the goal to develop a globally concerted approach to actively monitor the safety of vaccines and immunization programs in pregnancy (20). The GAIA group utilizes the format of the Brighton Collaboration to assess safety outcomes in mothers and infants after maternal vaccination, determining the level of certainty in the assessment of the event, to ensure uniformity and comparability in different settings. In addition to pertinent clinical case definitions, the GAIA consortium also published guidelines and tools for the assessment of safety of vaccines in maternal immunization clinical trials (47, 48). These guidelines were supported by the Global Advisory Committee on Vaccine Safety of the WHO (21), and various clinical case definitions are undergoing evaluation and validation as they are utilized in various settings from retrospective, to observational and prospective clinical trials worldwide.

## Conclusion

Maternal immunization has the potential to significantly improve maternal and child health worldwide by reducing maternal and infant morbidity and mortality associated with disease caused by pathogens that are particularly relevant in the perinatal period and in early life, and for which no alternative effective preventive strategies exist. Active research encompassing all aspects related to vaccines for administration during pregnancy is underway, with support of multiple stakeholders and global participation. Substantial progress has been made, and the availability of new vaccines licensed for use in pregnant women is an achievable goal. While many challenges remain to be addressed, the achievements in maternal immunization research to date have advanced the field and the prospects to make maternal immunization a feasible and accessible strategy to improve global health.

## Author Contributions

FM was responsible for designing and writing this article.

## Conflict of Interest Statement

The author declares no personal, professional, or financial relationships that could potentially be construed as a potential conflict of interest with the submitted work.
